# Initial symptoms and three months follow-up after acute COVID-19 in outpatients: An international prospective cohort study

**DOI:** 10.1080/13814788.2022.2154074

**Published:** 2023-01-19

**Authors:** Katarina Hedin, Alike W. van der Velden, Malene Plejdrup Hansen, Anna B. Moberg, Anca Balan, Pascale Bruno, Samuel Coenen, Eskild Johansen, Anna Kowalczyk, Peter Konstantin Kurotschka, Sanne R. van der Linde, Lile Malania, Jörn Rohde, Jan Verbakel, Heike Vornhagen, Akke Vellinga

**Affiliations:** a Futurum, Region Jönköping County, and Department of Health, Medicine and Caring Sciences, Linköping University, Linköping, Sweden; b Department of Clinical Sciences in Malmö, Family Medicine, Lund University, Malmö, Sweden; c Julius Center of Health Sciences and Primary Care, University Medical Center Utrecht, Utrecht, the Netherlands; d Center for General Practice at, Aalborg University, Aalborg, Denmark; e Department of Health, Medicine and Caring Sciences, Linköping University, Linköping, Sweden; f Balan Medfarm SRL, Cluj Napoca, Romania; g Département de Santé Publique, Université Côte d’Azur, Centre Hospitalier Universitaire de Nice, Nice, France; h Centre for General Practice, Department of Family Medicine & Population Health, University of Antwerp, Antwerp (Wilrijk), Belgium; i Centre for Family and Community Medicine, the Faculty of Health Sciences, the Medical University of Lodz, Lodz, Poland; j Department of General Practice, University Hospital Würzburg, Würzburg, Germany; k National Center for Disease Control and Public Health, Tbilisi, Georgia; l EPI-Centre, Department of Public Health and Primary Care, KU Leuven, Leuven, Belgium; m Nuffield Department of Primary Care Health Sciences, University of Oxford, Oxford, UK; n Data Science Institute, National University of Galway, Galway, Ireland; o School of Public Health, Physiotherapy and Sports Science, University College Dublin, Dublin, Ireland

**Keywords:** COVID-19, primary care, lingering symptoms, daily activities, post-COVID, long-COVID

## Abstract

**Background:**

Most studies on long-term follow-up of patients with COVID-19 focused on hospitalised patients. No prospective study with structured follow-up has been performed in non-hospitalised patients with COVID-19.

**Objectives:**

To assess long-COVID and post-COVID (WHO definition: symptomatic at least 12 weeks), describe lingering symptoms, their impact on daily activities, and general practice visits and explore risk factors for symptom duration in outpatients.

**Methods:**

A prospective study of adult outpatients with confirmed SARS-CoV-2 infection and symptoms consistent with COVID-19 in 11 European countries, recruited during 2020 and 2021 from primary care and the community. Structured follow-up by phone interviews (symptom rating, symptom impact on daily activities and general practice visits) was performed at weeks 2, 4, 8, and 12 by study personnel. Data was analysed descriptively by using correlation matrixes and Cox regression.

**Results:**

Of 270 enrolled patients, 52% developed long-COVID and 32% post-COVID-syndrome. When only considering the presence of moderate or (very) severe symptoms at weeks 8 and 12, these percentages were 28% and 18%, respectively. Fatigue was the most often reported symptom during follow-up. The impact of lingering symptoms was most evident in sports and household activities. About half (53%) had at least one general practice contact during follow-up. Obese patients took twice as long to return to usual health (HR: 0.5, 95%CI: 0.3–0.8); no other risk profile could predict lingering symptoms.

**Conclusion:**

Long-COVID and post-COVID are also common in outpatients. In 32%, it takes more than 12 weeks to return to usual health.

KEY MESSAGES

- One out of three COVID-19 outpatients still experiences symptoms at 12 weeks, 18% experience moderate to (very) severe symptoms.
- About half of patients had at least one general practice contact for COVID-19-related symptoms during these 12 weeks.
- Apart from obesity, no other clear risk profile could predict lingering symptoms.

## Introduction

The ongoing pandemic caused by SARS-CoV-2 remains an international public health emergency and has resulted in enormous global morbidity and mortality. Before the new Omicron variant spread by September 2021, more than 200 million COVID-19 cases and 4.3 million deaths were recorded globally (WHO dashboard) [1].

According to the WHO definition, a symptomatic COVID-19 case is a person who has developed signs and symptoms suggestive of COVID-19, including fever, cough, fatigue, anorexia, shortness of breath, myalgia and gastrointestinal manifestations [2]. Loss of smell and/or taste is also reported, as well as various mental and neurological symptoms [3]. Mild COVID-19 illness is defined as an uncomplicated respiratory tract infection with non-specific symptoms, including fever and cough and moderate illness as pneumonia without the need for supplementary oxygen [2]. About 80% of patients with COVID-19 have mild or moderate disease, and self-manage or are managed by their general practitioner (GP); up to 6% require hospital admission [4,5].

According to the WHO, long-COVID is defined as still experiencing symptoms 2 months after a SARS-CoV-2 infection, and post-COVID syndrome as symptoms for longer than 3 months which cannot be explained by an alternative diagnosis [6].

A systematic review of post-COVID-19 cases showed that more than half experienced symptoms 6 months after recovery, including functional mobility impairments, pulmonary abnormalities, and mental health disorders [7]. However, 80% of included patients were recruited in hospital and cases were retrospectively evaluated [7]. A recent study of patient data extracted from general practice records included nearly half a million patients with a COVID-19 diagnosis, with the most common complaints after 4 weeks being joint pain (2.5%) and anxiety (1.2%) [4].

To our knowledge, no prospective study with structured follow-up of the repertoire of COVID-19 symptoms at set times has been performed in outpatients. Therefore, we set out to determine the prevalence of long-COVID and post-COVID, to identify lingering symptoms and assess their impact on daily activities and number of general practice visits in adult outpatients with COVID-19. In addition, we explored risk factors related to a longer time to return to usual health.

## Methods

We set up a European, prospective, exploratory study of adult outpatients recruited from primary care and the community with laboratory-confirmed SARS-CoV-2 infection and symptoms consistent with COVID-19. National research teams were invited through the General Practice Research on Infections Network (GRIN, https://www.grinweb.org/) and the VALUE-Dx Primary Care Network (https://www.value-dx.eu) [8]. Each research team included a minimum of 10 to a maximum of 30 patients.

### Patient recruitment

Patients were informed by their GP, primary care nurse, or practice assistant when they contacted or consulted the practice for their symptoms *via* a leaflet in testing centres, through the research team’s personal contacts and *via* snowballing (relatives, housemates and friends of recruited patients). Patients were asked to contact the research team. Patients were asked to provide verbal informed consent during the first phone call, which was confirmed by a signed informed consent form.

The inclusion criteria were: laboratory-confirmed SARS-CoV-2 infection (PCR), symptoms consistent with COVID-19, maximum of 1 week since a positive test, age 18 years or older, able and willing to provide informed consent and to comply with phone calls. Patients hospitalised due to COVID-19 were excluded.

### Data collection

After patient contact was established, the first phone interview was performed. Patients were included from October 2020 until June 2021 in Belgium (Antwerp and Leuven), Denmark, France, Germany, Georgia, Ireland, Italy, the Netherlands, Poland, Romania, and Sweden. Upon inclusion, information on age, sex, weight/height, smoking and comorbidity defined as chronic respiratory condition (e.g. asthma, COPD, CF), diabetes, cardiovascular disease or ‘other’ was obtained. Furthermore, the presence of COVID-19 symptoms (fever, cough, shortness of breath, sore throat, fatigue/tiredness, headache, loss of appetite, gastrointestinal manifestation, myalgia, impaired taste, impaired smell, mental symptoms, emotional sensitivity), was captured.

All patients received follow-up calls at weeks 2, 4, 8, and 12 from study personnel. Outside the scope of this manuscript, after 12 weeks, monthly follow-up was done until symptom resolution up to a maximum of 1 year. Patients were asked to rate their symptoms as: absent, very mild, mild, moderate, severe, or very severe. Impact of symptoms on daily activities (work/education, income, caring for (grand-) children, household activities, sports, social life, non-sport hobbies) was rated as: no, slight, moderate, quite a bit, or severe.

Data was collected in a secured web-based tool (SurveyXact) hosted by Aalborg University Denmark, only including the patient study ID. Each research team kept patient’s name, phone number and study ID separately and secured until follow-up was completed.

A convenience sample of 250 patients was arbitrarily considered appropriate to answer the research question. This would allow for loss of follow-up of 10% and result in 75 patients (one-third expected) experiencing lingering symptoms at week 12 for analysis.

### Study endpoints

The primary endpoints of the study were the prevalence of long-COVID (defined as still symptomatic 8 weeks after a SARS-CoV-2 infection) and post-COVID (defined as symptomatic for 12 weeks, or longer, which an alternative diagnosis cannot explain) in outpatients. Secondary endpoints were duration of COVID-19-related symptoms (measured at 2, 4, 8 and 12 weeks), how these impacted patients ‘daily activities’ and general practice visits, and risk factors (e.g. age, sex, body mass index (BMI), and comorbidity) related to time to return to usual health.

### Statistical methods

Categorical data are presented as numbers and proportions and continuous data as medians with lowest and highest values. To visualise correlations between symptoms present at weeks 2 and 12, and correlations between individual symptoms and daily activity impact present at week 12, correlation matrices were generated (including correlation coefficients) in Tableau Professional Desktop Edition (2021.4.3).

A Cox regression was performed for the composite outcome ‘health returned to usual.’ Patients were considered to reach this outcome at the moment they did not report any more symptoms or the week they confirmed a return to normal health. Patients who did not fall in either category at week 12 were censored. A backward regression using presence of each symptom at baseline, sex, age, any comorbidity, as well as separately each individual comorbidity, overweight and obesity, was performed. Sex and age were kept in the model, but other variables remained if significant. *p-*Values < 0.05 were considered significant. Results are presented as hazard ratios (HR) with 95% confidence intervals (95%CI). Data analysis was performed using SPSS v27.0 software (IBM, Armonk, NY).

## Results

**Figure 1.**
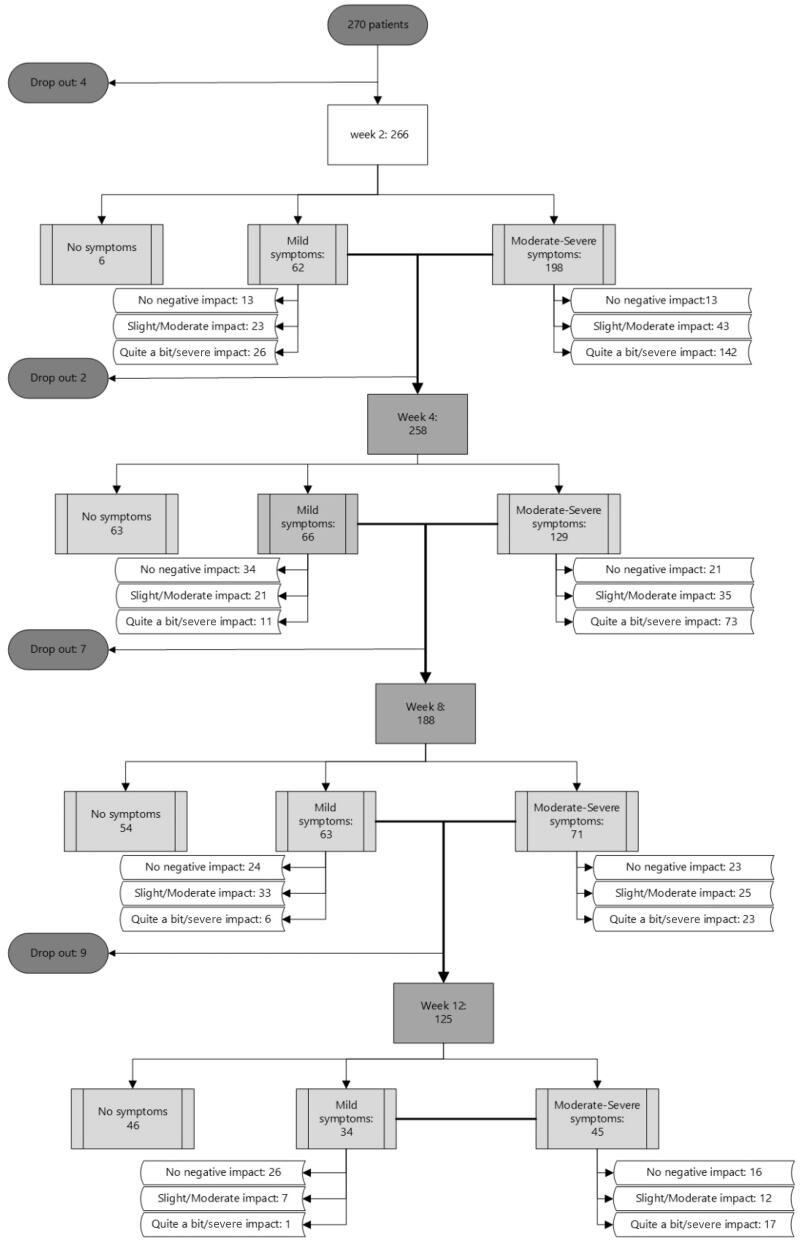
Patient flow with symptom severity and impact on daily activities during follow-up. Patients (absolute numbers are shown) are divided per follow-up moment with respect to symptom resolution and those still symptomatic to severity of symptoms. Total numbers of patients in the study: 266 at week 2, 264 at week 4, 257 at week 8, and 248 at week 12.

A total of 270 outpatients were included: Belgium Antwerp (*n* = 30), Belgium Leuven (*n* = 27), Denmark (*n* = 21), France (*n* = 17), Germany (*n* = 30), Georgia (*n* = 20), Ireland (*n* = 20), Italy (*n* = 23), Netherlands (*n* = 20), Poland (*n* = 14), Romania (*n* = 18) and Sweden (*n* = 30). A total of 22 patients were lost to follow-up (Figure 1), and there were no missing data for the remainder due to the follow-up phone calls. Baseline characteristics of the included patients are presented in Table 1. Comorbidities were reported by 45% of patients, most often cardiovascular disease (21%). BMI was normal for 49% (BMI: 18.5–24.9), 2% were underweight (BMI: < 18.5), 31% overweight (BMI: 25–29.9) and 18% obese (BMI: ≥ 30).

**Table 1.** Characteristics of COVID-19 outpatients at inclusion.

Patient characteristics	*n* = 270
Female, *n* (%)	161 (60)
Age, years, median (min–max)	50 (18–83)
BMI, kg/m^2^, median (min–max)	25 (16–45)
Current smoker, *n* (%)	37 (13.7)
Any comorbidity, *n* (%)	121 (45)
Any chronic respiratory condition*	20 (7.4)
Diabetes	14 (5.2)
Cardiovascular disease, including hypertension	57 (21.1)
Other^#^	47 (17.4)

*Asthma, COPD or cystic fibrosis, ^#^allergy/eczema, psychiatric disorder, breast cancer, lung cancer, hyperthyroidism, rheumatic disease, psoriasis were reported.

### Patient flow with symptoms severity and impact on daily activities during follow-up

Figure 1 shows the patient flow, with symptom severity and impact of symptoms on daily activities during the 12 weeks of follow-up. The proportion of patients reporting any COVID-19-related symptom decreased from 98% (260/266) at week 2, to 74% (195/264) at week 4, 52% (134/257) at week 8 (long-COVID) and 32% (79/248) at week 12 (post-COVID). Considering only the presence of moderate to (very) severe symptoms at week 8 and 12, the proportions decreased to 28% (71/257), and 18% (45/248), respectively. Of those patients reporting symptoms, 65% (168/260) experienced ‘quite a bit’ to ‘severe’ impact of their symptoms on at least one of the questioned daily activities at week 2, 43% (84/195) at week 4, 22% (29/134) at week 8 and 23% (18/79) at week 12.

### Specification of COVID-19-related symptoms during the first 12 weeks

**Figure 2.**
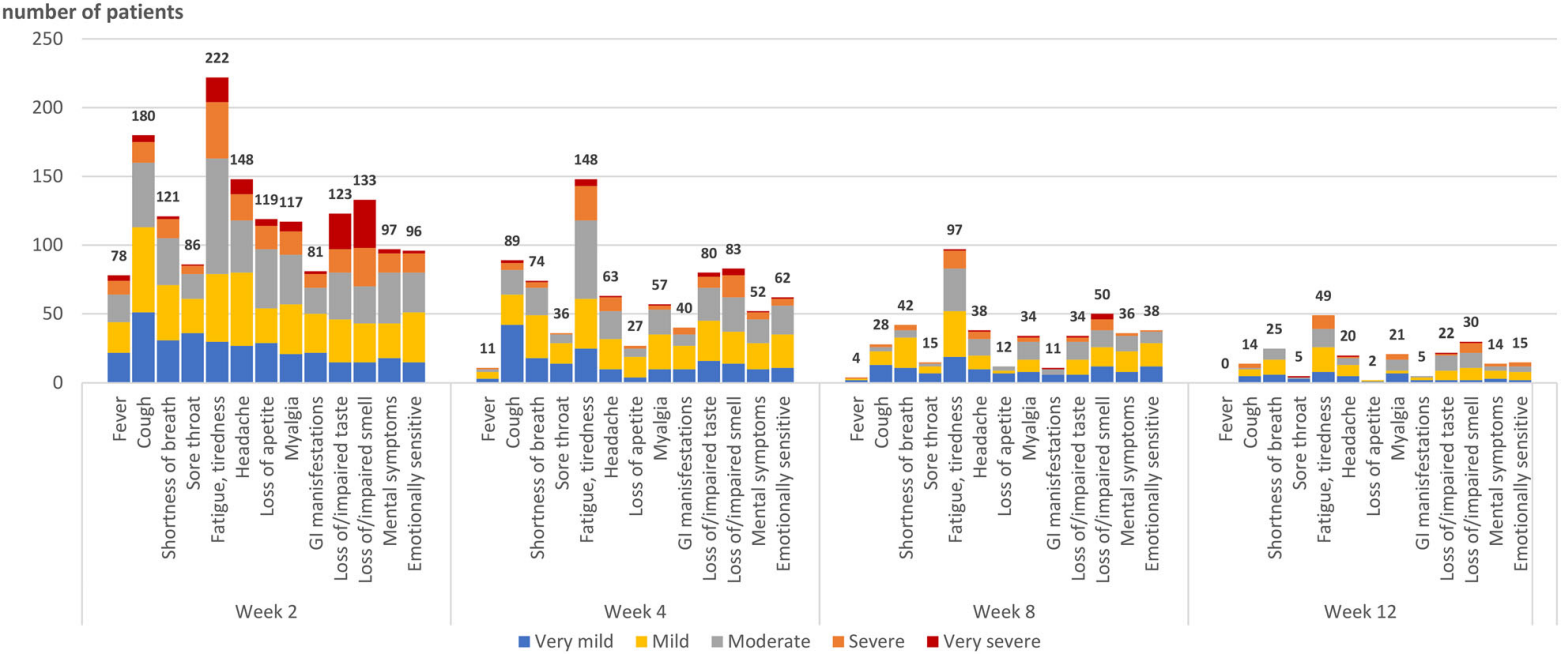
Frequency and severity of self-reported symptoms in COVID-19 outpatients over 12 weeks after a positive SARS-CoV-2 test. Absolute numbers of patients reporting each individual symptom are shown, with severity distribution.

At inclusion, fatigue/tiredness was the most often reported symptom (79%, 213/270), followed by headache (73%, 196/270) and cough (67%, 180/270). Figure 2 shows the trend of reported symptoms and their severities from week 2 to week 12. At week 2, fatigue, cough, loss of/impaired taste and/or smell and headache were most often reported and with highest severity. At week 12, fatigue (20%, 49/248) and loss of smell (17%, 30/248) remained the most often reported symptoms, of whom, respectively, 20% and 27% rated these symptoms as (very) severe. Additionally, myalgia, headache and shortness of breath persisted until week 12 in about 9% of patients.

**Figure 3.**
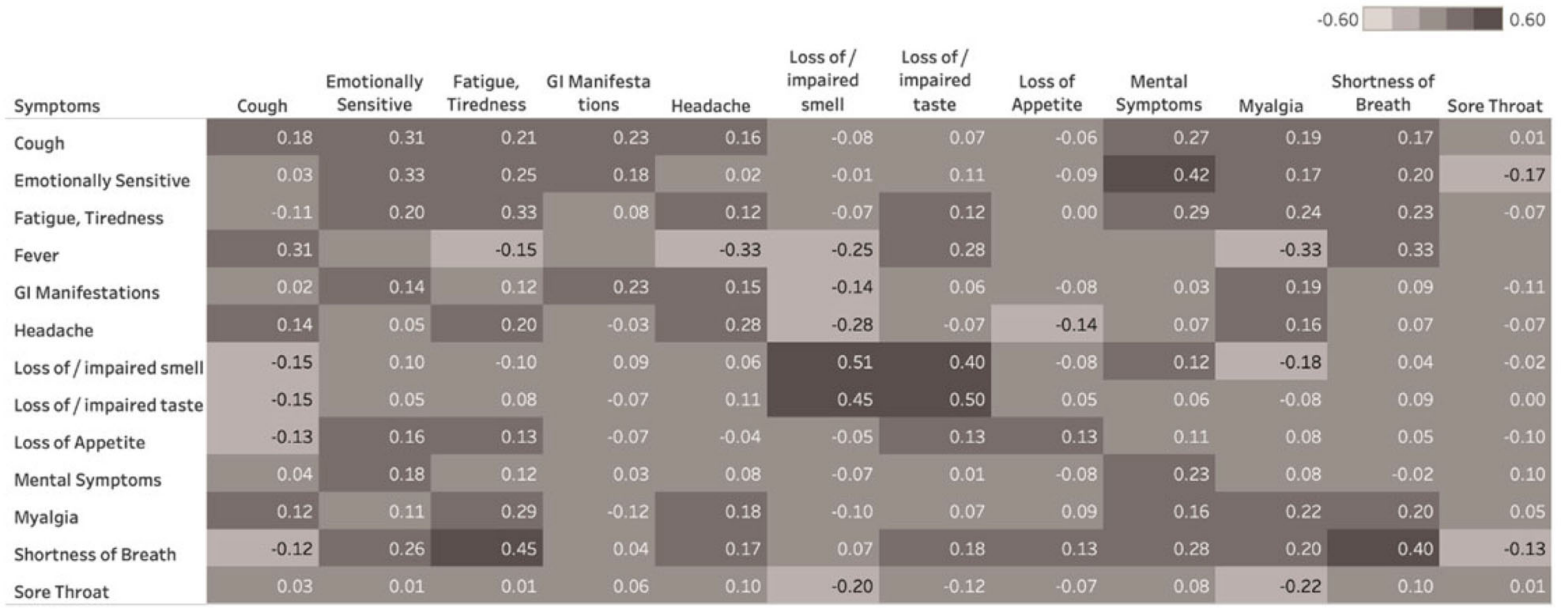
Correlation of COVID-19-related symptoms between week 2 and week 12. Univariate correlation between presence and severity of symptoms at week 2 (column) and week 12 (row). Darker tones correspond to stronger correlations. Empty cells indicate no patients with that particular symptom combination at week 2 and 12.

Figure 3 displays the correlation between the presence of individual symptoms at week 2 and week 12, showing correlations for individual symptoms but in particular for shortness of breath and loss of/impaired taste and/or smell. Furthermore, it suggests a long-term effect of early reported shortness of breath on fatigue, mental symptoms and emotional sensitivity.

### Specification of impact of symptoms on daily activities

Table 2 shows that lingering symptoms of COVID-19 negatively impacted most daily activities. Initially, education/work, household activities and social life were most often reported to be affected. Continued impact of symptoms was most evidents for sports, followed by education/work and household activities.

**Table 2.** Impact on daily activities reported by outpatients with lingering COVID-19-related symptoms.

		Week 2	Week 4	Week 8	Week 12
		*n* = 260	*n* = 195	*n* = 134	*n* = 79
		*n* (%)	*n* (%)	*n* (%)	*n* (%)
Education/work	Any impact	154 (59.2)	95 (48.7)	47 (35.0)	17 (21.5)
	Quite a bit/severe	92 (35.3)	38 (19.5)	15 (11.2)	5 (6.3)
Income	Any impact	72 (27.7)	35 (17.9)	22 (16.4)	4 (5.1)
	Quite a bit/severe	38 (14.6)	16 (8.2)	6 (4.5)	1 (1.3)
Caring for (grand) children	Any impact	70 (26.9)	42 (21.5)	24 (17.9)	10 (12.7)
	Quite a bit/severe	42 (16.2)	13 (18.5)	3 (2.2)	2 (2.6)
Household activity	Any impact	171 (65.8)	86 (44.1)	46 (34.3)	26 (20.3)
	Quite a bit/severe	84 (32.3)	26 (13.3)	5 (3.7)	7 (8.9)
Sports	Any impact	151 (58.1)	91 (46.7)	54 (40.3)	37 (46.8)
	Quite a bit/severe	95 (36.5)	46 (23.5)	16 (12.1)	12 (15.2)
Social life	Any impact	161 (61.9)	80 (41.0)	40 (29.9)	15 (19.0)
	Quite a bit/severe	108 (41.5)	33 (16.9)	11 (8.2)	2 (2.6)
Hobbies – non-sport	Any impact	114 (43.8)	52 (26.7)	30 (22.4)	14 (17.7)
	Quite a bit/severe	66 (25.3)	17 (8.8)	5 (3.7)	4 (5.0)

Any impact refers to the responses slight, moderate, quite a bit, or severe; quite a bit and severe are also presented separately. Absolute numbers and percentages of patients reporting lingering COVID-19-related symptoms at weeks 2, 4, 8 and 12 after a positive SARS-CoV-2 test.

**Figure 4.**
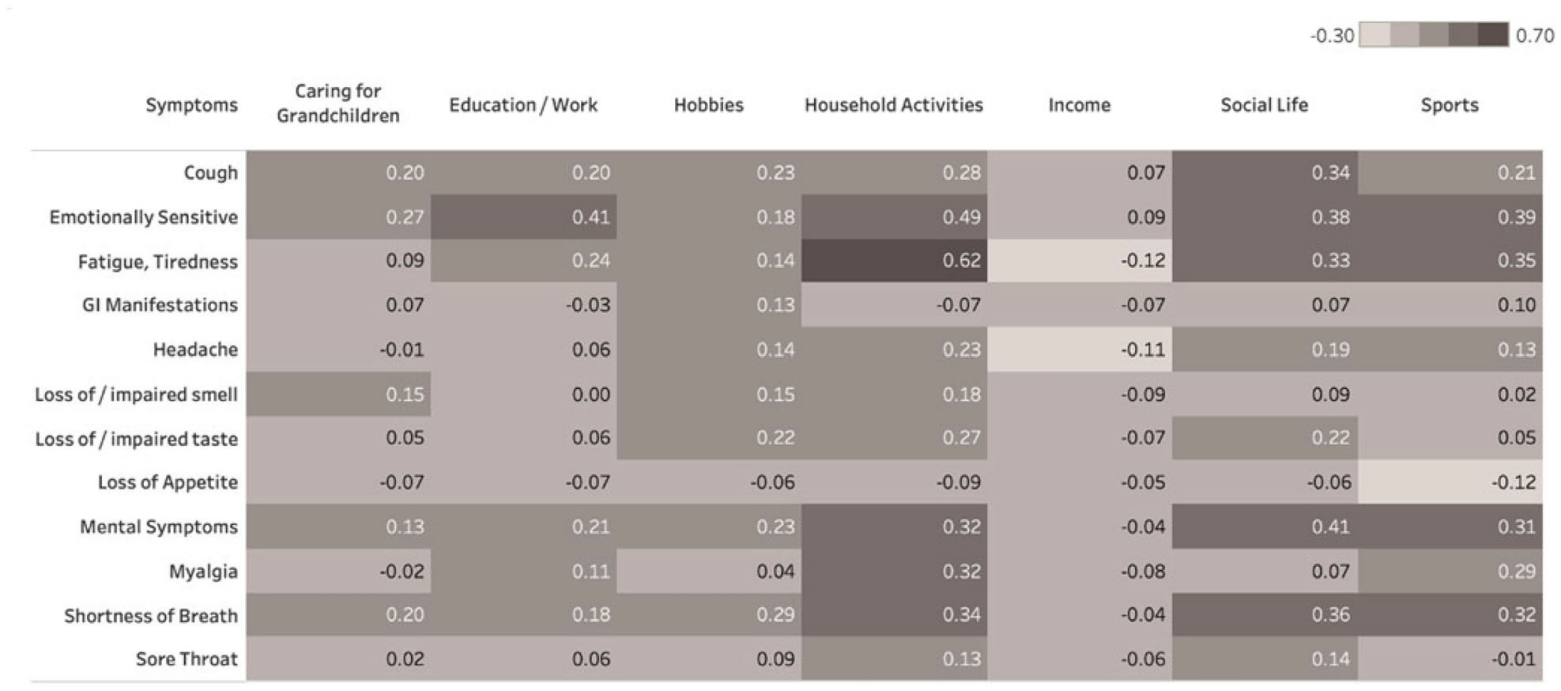
Correlations between COVID-19-related symptoms at week 2 and their impact on daily life variables at week 12. Univariate correlations between presence and severity of symptoms week 2 (column) and impact on daily life at week 12 (row). Darker tones correspond to stronger correlation.

Figure 4 shows the correlation between individual symptoms at week 2 and daily activities’ impact at week 12. At week 12, most severe impact was on household activities, social life and sports. Fatigue, mental symptoms, emotional sensitivity and shortness of breath at week 2 showed the strongest correlations with daily activities impact at week 12.

### Patient factors and symptoms related to time to return to usual health

**Figure 5.**
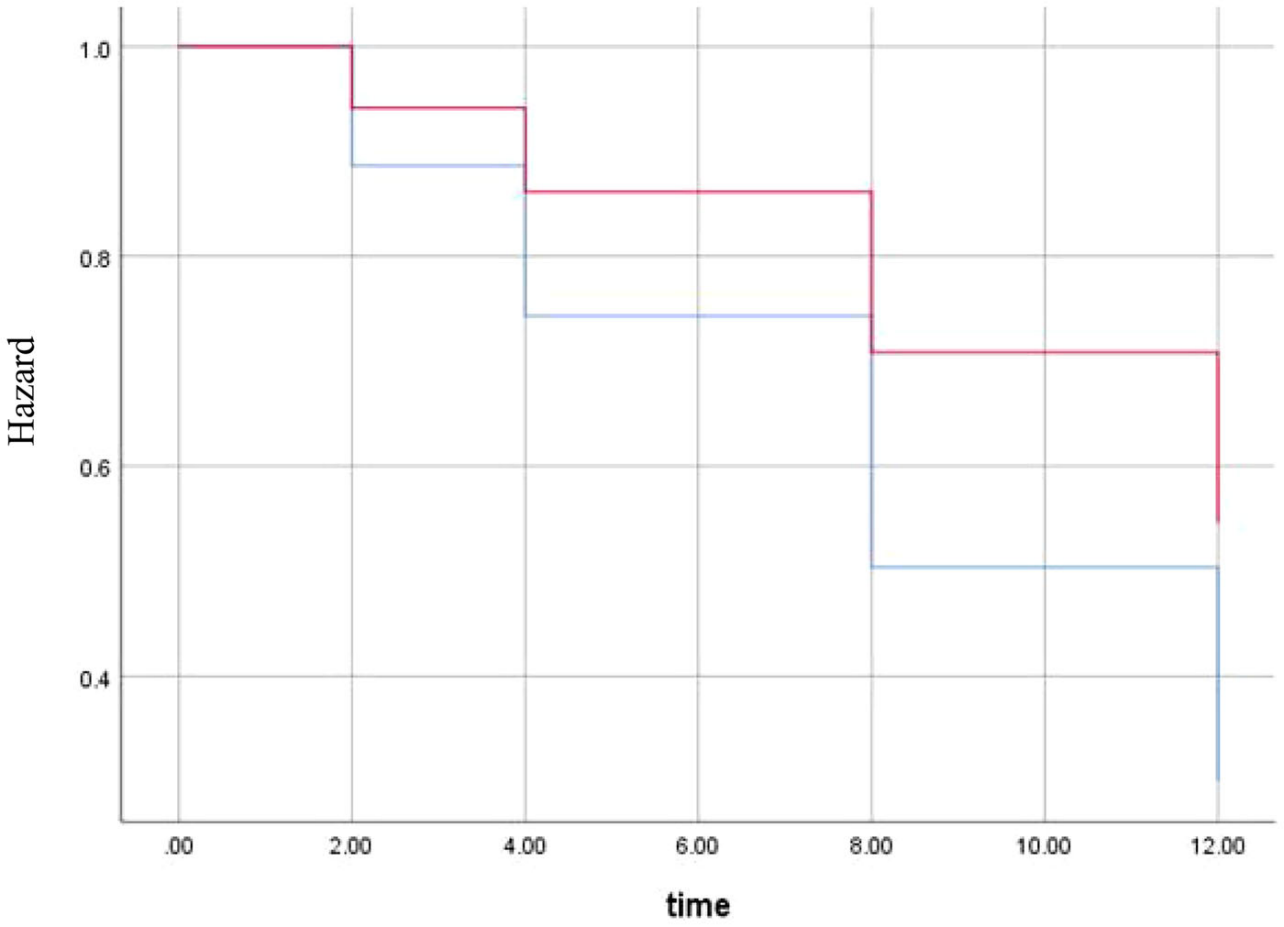
Cox regression for time to return to usual health for obese patients compared to non-obese. Graph shows the hazard for obese patients (red) and non-obese patients (blue). Outcomes were adjusted for age, sex, sore throat and gastrointestinal manifestations (variables included in the final model, *p*-value = 0.007). *Y*-axis starts at 0.3 to better visualise the difference between lines. The *X*-axis shows number of weeks.

The Cox regression with time to return to usual health showed a significant HR for obesity (HR: 0.5, 95%CI: 0.3–0.8), sore throat (HR: 0.7, 95%CI: 0.5–0.97) and gastrointestinal manifestations (HR: 0.7, 95%CI: 0.5–0.9). The HR for obesity can be interpreted as doubling the risk of post-COVID when obese (Figure 5). No other patient characteristics were significantly associated with longer return to usual health.

### General practice visits related to COVID-19

Over half of all included patients (53%) had at least one contact with a GP or nurse for COVID-19-related symptoms during the 12 weeks of follow-up. Overall, 16% of patients consulted their general practice three or more times. The median value of contacts was 1 (min: 0–max: 10). Of those patients still symptomatic at week 12 (*n* = 79), 20% consulted their general practice three or more times.

## Discussion

### Main findings

This prospective, exploratory study of adult outpatients with SARS-CoV-2 infection performed in eleven European countries shows that 52% can be considered to have long-COVID (symptomatic at week 8) and 32% post-COVID (WHO definition: symptomatic at week 12). When only considering the presence of any symptom rated as moderate or (very) severe at weeks 8 and 12, these percentages decrease to 28% and 18%, respectively. During the first 12 weeks after a positive test, fatigue was the most often reported symptom, and sports, education/work and household activities were most often affected. Obese patients appeared to take twice as long to return to usual health. Apart from sore throat and gastrointestinal manifestations at baseline (weak association), no other relevant risk factors could be identified.

### Comparison with existing literature: Return to usual health

Compared to a US outpatient cohort, which reported 35% not returned to usual health 3 weeks after their positive test, our cohort showed that 74% had not returned to usual health at week 4 [9]. Data obtained through the COVID Symptom Study app, in which individuals self-reported their symptoms prospectively, showed lower estimates of 13% with symptoms at 4 weeks, 4.5% at 8 weeks and 2.3% at 12 weeks [10]. However, inclusion characteristics, setting, number and items questioned, and the definition of ‘return to usual health’ differed between studies.

### Comparison with existing literature: Lingering symptoms

We found fatigue/tiredness the most common symptom at weeks 8 and 12, followed by shortness of breath and slowly recovering loss of smell. A study of long-COVID patients also identified these symptoms with fatigue, post-exertional malaise and cognitive dysfunction, the most frequently reported symptoms after 6 months [11].

Ongoing symptoms, particularly fatigue and shortness of breath, impacted daily activities in 15% of our cohort at 12 weeks. Even though lingering symptoms may be mild, ongoing impact and lack of a total return to usual health is relatively high. This impact can also be felt economically, for instance in work absenteeism and health care costs; three or more general practice visits for symptoms related to COVID-19 were reported by one in six patients (16%).

### Comparison with existing literature: Predictors of time to return to usual health

No clear clinical profile predicted a longer time to return to usual health, even though gastrointestinal manifestations and sore throat as initial symptoms were weakly associated. However, one consistent finding is that obesity is a strong predictor, appearing to double the chances of post-COVID, similar to other studies [9]. Our study did not identify other (chronic) conditions but other studies have specified depression and asthma to impact symptom duration [10]. Age was not a significant predictor in our study, which may be due to the wide range of ages. Other studies may have introduced bias in recruitment of certain age groups when using digital modes of recording (COVID Symptom Study app) or due to recruitment of more severe and often older patients. Similar to our study, the COVID Symptom Study app also found the co-occurrence of anosmia (loss of smell) and ageusia (loss of taste) associated with duration of symptoms, which is most likely just due to slow recovery and not necessarily a risk factor for prolonged symptom duration [12].

### Strengths and limitations

The strength of our study is the prospective data capture using a structured questionnaire, with regular long-term follow-up and minimal loss to follow-up. We included outpatients from many countries, healthcare systems, ages, and cultural backgrounds, with representative comorbidity prevalences [8]. At the time of patient inclusion, routine PCR-based testing was implemented in all participating countries. Patients were recruited after they contacted or consulted the GP *via* testing centres and from the community to capture a broad case-mix, independent of thresholds for consulting the GP.

However, this is a convenience sample of COVID-19 outpatients recruited *via* different pathways. One can imagine that patients seeking help from their GP were more severely ill at baseline and, therefore, potentially prone to longer symptom duration. Also, our study’s total number of patients was relatively small (*n* = 270). Studies with higher numbers have found more correlations in their data but still did not identify a clear risk profile with underlying conditions beyond obesity. In addition, we did not include information on the patients’ physical and mental health before COVID-19.

For generalisability, our study included patients before the emergence of the omicron variant, assuming that mainly delta dominated our cohort [13]. The omicron variant is reported to be less severe and, therefore, expected to result in less long-COVID [14]. Additionally, we can assume that the vast majority of patients in our cohort were unvaccinated, as vaccination only became available at the end of the study’s inclusion period. Our findings, therefore, mostly apply to unvaccinated patients. With vaccinated people less likely to report symptoms of long-COVID, some studies estimate that vaccination halves the risk of long-COVID; our estimates of long-COVID may be higher than prevalent in a vaccinated population [12,15].

Nevertheless, this cohort will address a gap in knowledge of outpatients [16]. In some countries, long-COVID clinics have been set up. Still, extensive prospective research with follow-up of a year into long-COVID and progress in identifying treatments as part of its COVID-19 therapeutics plan has not been reported [16].

## Conclusion

Long-COVID and post-COVID are common in predominantly unvaccinated outpatients. One in four reports moderate to (very) severe symptoms 8 weeks after a positive SARS-CoV-2 test, one in 6 after 12 weeks. Predicting a longer return to usual health remains difficult as, apart from obesity, no apparent risk profile appeared. The strong association of obesity with a longer return to usual health may be an indication for targeted vaccination and requires further study.
